# Impact of Hot Water Extraction on the Compaction Efficiency and Material Properties of *Miscanthus giganteus* in Pellet Production

**DOI:** 10.3390/ma17246137

**Published:** 2024-12-15

**Authors:** Kamil Roman, Julia Dasiewicz, Monika Marchwicka

**Affiliations:** 1 Institute of Wood Sciences and Furniture, Warsaw University of Life Sciences–SGGW, 02-787 Warsaw, Poland; kamil_roman@sggw.edu.pl (K.R.); monika_marchwicka@sggw.edu.pl (M.M.); 2 Faculty of Wood Technology, Warsaw University of Life Sciences–SGGW, 02-787 Warsaw, Poland

**Keywords:** *Miscanthus giganteus*, pellets, renewable energy, hot water extraction, energy

## Abstract

The present study aimed to determine the effect of material modification by hot water extraction (HWE) on the compaction efficiency of shredded *Miscanthus giganteus* stalks in the pellet production process. Samples were prepared to differ in the number of HWE cycles: HWE I was subjected to a single cycle, HWE II was subjected to two cycles, and HWE III was subjected to three cycles and unmodified material. An analysis of the compaction process was carried out to evaluate the effect of HWE on density and energy consumption. In this study, HWE pre-treatment was applied to improve biomass compaction efficiency and material properties, improving biofuel production’s sustainability and efficiency. A small change was found in glucose and xylose content, suggesting that HWE slightly affects these sugars. It was found that HWE significantly increased the density of compacted *Miscanthus giganteus* biomass, with a peak density of 388.7 kg·m^−3^ after the first HWE cycle. Ash content was reduced by 50% after one HWE cycle, making the biomass more suitable for biofuel applications. Furthermore, pretreatment with HWE reduced energy consumption during compaction, enhancing process efficiency. This study highlights the role of hot water extraction (HWE) in improving miscanthus compaction, enhancing density, reducing ash, and lowering energy use.

## 1. Introduction

Conventional fossil fuels remain the primary source of energy supply as the demand continues to increase. Reducing greenhouse gas emissions is essential as fossil fuel supplies decline and their effects on the environment increase. As a result, the use of biomass for power and heat has increased significantly, prompting interest in pelletization and briquetting [1]. An effective way may be to replace fossil fuels with energy crops, which are known to be a component of renewable bioenergy fuel sources [1,2]. That is why looking for suitable energy plants is very important now to meet the ever-increasing demand for energy [1]. In addition to lowering greenhouse gas emissions, energy crops may potentially be able to store carbon dioxide [3,4,5]. For civilization to remain sustainable, it is crucial to find appropriate energy crops to meet the growing need for energy.

Renewable energy sources, which are environmentally friendly and valuable in reducing greenhouse gas emissions, help combat climate change [6,7]. They can provide more affordable and easily accessible energy and open up local opportunities for socioeconomic development [8]. Since renewable resources never run out, the world’s energy needs can be met repeatedly [5]. Developing renewable energy sources is necessary not only for environmental protection but also to create new economic and social opportunities. Investment in renewable energy sources is key to ensuring sustainable development and improving the quality of life for future generations.

A significant renewable energy source is agricultural solid biomass, a plant product. It mainly comprises agro-industrial waste like seed husks, energy crops like miscanthus cultivated especially for energy, and crop leftovers like straw or husks. Additionally, waste from trimming fruit trees and vines are included in agricultural biomass [1,2,9,10]. The biomass from agriculture may also contain residues such as corn stalks, corn cobs, straw, sunflower stalks, sunflower husks, fruit pits, pruning residues from grapevines, and fruit trees [7,8]. Biomass from crops, such as *Miscanthus giganteus*, is a renewable energy source with a high yield and adaptability to various environmental conditions. Compaction technologies and pretreatment methods enable this crop, known for its resilience and ecological benefits, to be optimized for biofuel production [1,2]. *Miscanthus giganteus* has a very high yield, is resilient, and adapts well to diverse climates. The crop grows well on marginal lands unsuitable for food production, making it an eco-friendly option for renewable energy. The C4 photosynthesis pathway promotes growth efficiency, while its high content of lignocellulosic matter makes it an ideal candidate for biofuel production [11]. This genus has 14 to 20 species, which may grow in various climates [12]. Despite its use as an ornamental grass, it is now considered one of the most promising energy crops [12,13]. Miscanthus is not only a clean, renewable energy source that can replace fossil fuels, but it can also be very helpful in ecological restoration and environmental cleanup [14,15]. In contaminated soil, miscanthus can decompose organic materials and accumulate heavy metals. Contaminants can be successfully eliminated from contaminated soil by planting it there. Miscanthus grass biomass harvested during the growing season has different functional properties than after the above-ground parts have dried out [16,17]. Differences include higher moisture content and different yield structures [18]. After undergoing this transformation, biomass feedstocks can be utilized as bioenergy feedstocks. Before the biomass can be used, it must be dried, which takes more time and energy than usual. Additionally, while the biomass is drying, it could become polluted with contaminants.

Reference [11] evaluates whether multi-cycle HWE can be applied to biofuel feedstock preparation to unlock new efficiencies. The lifespan of this plant reaches up to about 16 years. *Miscanthus giganteus* shows physiological and ecological benefits due to its multi-year life cycle. In its rhizomes, nutrients are recycled and stored during periods of no growth [19]. In Poland, miscanthus was cultivated on about 2000 ha in from 2009 to 2011; by 2013, the area decreased to 733 ha, and it may now be even less than 500 ha. These data were provided by the Polish Biomass Association (POLBIOM, Warsaw) [20]. The cost of producing miscanthus biomass is very high, which may influence a low desire to grow it in Poland. Despite limited cultivation in some regions, its potential for meeting growing energy demands is evident. *Miscanthus giganteus* is being adopted as a mainstream bioenergy crop because of this study, which illustrates its economic and environmental benefits.

The briquetting of plant material has been thoroughly discussed in the literature [6,21,22] and has been characterized as the reactions occurring during the compaction process in a reciprocating flow briquetting machine [6,21,22]. Plant materials compaction reveals a sophisticated style of functioning for the complete system. A plunger cycles through the material in the machine’s working chamber before compressing it again in the compaction chamber. Due to the compaction backfill reaction forces, the compacted material experiences an opposing pressing force throughout each cycle. The briquette is expelled through the guides, stabilizing its structure once the resistance of the resultant force (arising from the response of the forces of the lagging briquette and friction) is exceeded.

The dimensions and the briquette press’s technical characteristics dictate the working pressure. In the literature, the ranges from several to over 100 MPa are used for the lignocellulosic compacting process [21]. This means that choosing the correct unit pressure for the briquetting process is a challenging and time-consuming process that is also dependent on the length and irregularity of the fraction (the degree of material fragmentation) and other process-related variables like the type of biomass and its moisture content [21,23]. Specific authors [21] suggest that wood waste should be briquette at a pressure of 45 to 60 MPa [21]. By reducing the ash content and increasing density, HWE improves compaction efficiency and reduces energy consumption during briquet-ting processes. Compaction methods, such as briquette and pelletizing, improve biomass transport and energy conversion efficiency. It is possible to transform the structural properties of *Miscanthus giganteus* to make it more viable as a renewable energy source. The multi-cycle HWE approach presented here represents a significant advancement in bioenergy pretreatment.

This article aimed to determine whether hot water extraction (HWE) treatment could effectively reduce the energy required during the densification process of lignocellulosic materials. If HWE does not achieve the desired reduction in energy use, an alternative approach worth exploring is using miscanthus as a non-liquid biofuel for energy production. Wood contains a variety of chemical compounds categorized by function into three groups: skeletal, encrusting, and companion compounds. Skeletal compounds, also known as holocelluloses, include cellulose and hemicelluloses. Encrusting compounds, such as lignin and pectin, contribute to structural rigidity. Companion compounds, including sugars, fats, and proteins, are present in living wood cells alongside other compounds like dyes, tannins, resins, alkaloids, glycosides, and minerals. Notably, minerals leave behind ash after combustion, making up about 1% of the dry weight of wood, primarily as calcium carbonate [9,24,25]. Cellulose, hemicellulose, and lignin content play a critical role in the suitability of lignocellulosic biomass for energy applications. This research provides novel insights into how HWE impacts chemical composition, reduces ash content, and improves the structural characteristics of *Miscanthus giganteus* for biofuel production.

Lignocellulosic materials, primarily cellulose and hemicellulose, are increasingly considered suitable feedstocks for biofuel synthesis, particularly bioethanol [1]. The biofuel synthesis process relies on lignocellulosic sugars obtained from cellulose and hemicellulose through hydrolysis. Cellulose, the principal component of lignocellulosic biomass, can be efficiently converted into sugars via chemical, enzymatic, or acid treatment, while hemicellulose provides additional sugars for biofuel production [2]. A notable example of effective lignocellulosic biomass use is the Brazilian sugarcane industry, which serves as a model for bioethanol production [1]. This example underscores the role of lignocellulosic materials in advancing sustainable global energy solutions as viable alternatives to fossil fuels. Integrating lignocellulosic biomass into biofuel production supports energy sustainability and reduces the dependency on non-renewable resources, further reinforcing its potential in renewable energy pathways.

A novel application of sequential hot water extraction (HWE) on *Miscanthus giganteus* is examined in this study, enhancing its potential as a sustainable and efficient biofuel source. As part of this study, HWE is investigated for its cumulative effects on the compounding properties of *Miscanthus giganteus*, including density, ash reduction, and energy efficiency, differentiating it from prior research that focused solely on single-cycle treatments.

## 2. Materials and Methods

### 2.1. Material Characteristics and Moisture Content

The material used in the study was *Miscanthus Gigantus*, a high-yielding energy crop shredded and prepared for compaction to evaluate its biofuel potential. The material parameters specified in the specification shall be used to prepare the raw material. Shredded miscanthus stalks were sorted and ground to a uniform particle size of 0.1 mm using a Hydrolab orbital shaker. This material was evaluated for compaction under controlled conditions. The type of substance utilized and how it was obtained probably showed signs of the residue’s initial makeup when it was first collected. The C.B.K.O Hydrolab (Warsaw, Poland) orbital shaker was used to sort the crushed material into fractions. Laboratory tests entailed controlled compaction of the physical and mechanical properties of the material, focusing on its performance during compaction. Hot water extraction (HWE) pretreatment enabled a comprehensive evaluation of miscanthus as a potential feedstock for advanced biofuel applications.

It was crucial to stabilize moisture content during the preparation process. Hot water extraction (HWE) treatment further modified the material’s physical and chemical properties, enhancing its compaction efficiency and reducing ash content. The moisture content of *Miscanthus Gigantus* samples was measured regularly by sampling the container at regular intervals. Every sample was dried in a laboratory chamber at 105 °C for 24 h after removal. Moisture content was standardized by placing the samples in a laboratory forge. Following two weeks of observation, the moisture content of the samples stabilized at 12%. After drying, the samples were weighed before and. The absolute moisture content was calculated as a percentage of their weight before and after drying. The accuracy of this method made it possible to determine the moisture distribution in the material. Following the literature’s guidelines, the required moisture level was maintained during the drying process. To ensure compliance with the applicable standards, the moisture content of crushed miscanthus samples was analyzed using the standards. An additional moisture analysis was performed on samples of five grams, randomly selected from the material before being placed into the condensation chamber using a Radwag MAC 50 moisture analyzer (Radom, Poland).

### 2.2. The Material HWE Treatment

The study used hot water extraction (HWE) treatment with specialized equipment to meet the experiment’s requirements. The equipment has three main components that work together. In the extraction process, distilled water was used to fill the bottom part of the reactor. At the top of the reactor, there was a part containing materials undergoing HWE treatment. Both parts were connected and equipped to the pressure discharge hole to maintain necessary pressure levels during extraction. The design of this apparatus ensured precise and consistent extraction conditions throughout the experiment. To ensure reliability, the HWE procedure itself was carefully planned and executed. Test samples were weighed to ensure they contained approximately 10 g of material. To ensure consistency across all samples, this precise measurement was essential. After the prepared material was weighed, it was placed in the container, where high-pressure and high-temperature conditions are inherent throughout the HWE process.

In the following step, 400 mL of distilled water was added to the bottom reactor. Throughout all experiments, this specific quantity of water was used. The water was heated to 100 °C within the reactor while the pressure was maintained at 2 MPa. The extraction temperature and pressure had to be monitored closely because any deviation could affect the outcome. The procedure was structured into three successive extraction cycles, each designed to test the cumulative effects of the extraction process. The first cycle of extraction consisted of a single round. Two consecutive extractions were performed in the second cycle, while three were performed in the third cycle. The extraction times were standardized to about 20 min, which is optimal for effective extraction without causing sample degradation. The material was removed and dried in labeled containers following each extraction cycle to ensure precise sample tracking. The distilled water used in the process was evaporated to measure precipitate mass, representing substances extracted during HWE. These steps supported the study’s objective of evaluating how multi-cycle HWE impacts material characteristics, such as ash content, density, and energy efficiency, providing insights into biofuel optimization through HWE pre-treatment.

### 2.3. Compaction Process

The analysis of the compaction process of miscanthus as a lignocellulosic material involved the modernization of the available testing machine. A prototype compaction head with auxiliary equipment was developed and constructed as a diagnostic component to simulate the environment during pressure agglomeration. Instron 3382 testing machine (Norwood, MA, USA) was used to conduct laboratory strength tests. It included the testing machine, strength plates, an auxiliary device, and a measuring computer running Instrom IX (Version 6) software. Tests were conducted to demonstrate the increasing compaction force with piston displacement. The prototype compaction head was used to compile the sample and determine the material’s mechanical properties. The test measured the pressure characteristics during the process to identify the flow stress created and the strain energy produced. The tests were performed according to standards, the international standard for these tests.

Several parameters were considered during the design of the head, including the features of the testing machine. The machine manufacturer set the upper limit of the piston pressure at 100 kN. A thorough system for the compaction head needed to be created. The device compacted the prepared material using a piston encased in a steel sleeve. The lower part of the head was sealed with a steel cover. The main goal of the measurements was to analyze anisotropic materials during agglomeration. The apparatus allowed a detailed analysis of characteristics occurring during material agglomeration based on piston displacement. Stress and displacement were measured using measuring sensors and the test setup. Compaction forces inside 62 mm long head chambers were measured at a frequency of 0.01 Hz by the integrated measuring unit of the testing machine. The gauges available for calibration were used to calibrate all components before measurements. The precision of measurement was determined by the accuracy of the measuring equipment and the calibration of the individual components.

### 2.4. Chemical Composition

#### 2.4.1. Ash Content

**Figure 1:**
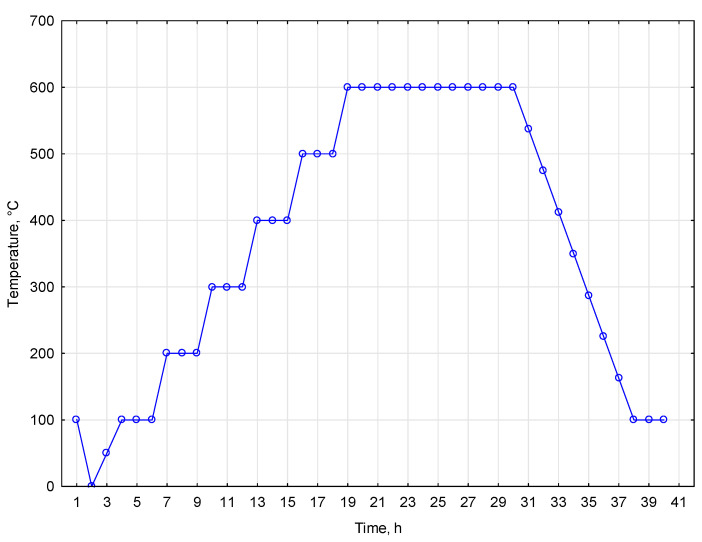
Temperature program for ash content determination.

The ash content of native miscanthus was determined using a laboratory muffle furnace to evaluate the biomass suitability for various applications. Initially, approximately 1 g of dried miscanthus samples was weighed accurately. The samples were subjected to an initial thermal treatment, heated to 100 °C, and held at that temperature for 1 h. This preliminary step was critical for evaporating moisture from the samples, ensuring that subsequent measurements reflected the accurate ash content. The samples underwent a series of incremental temperature increases following the initial heating. The first cycle involved heating the samples to 200 °C for 1 h, facilitating the decomposition of organic materials. This procedure was repeated at 300 °C, 400 °C, and 500 °C, with each heating step lasting 1 h. Each temperature increment allowed for the progressive combustion of residual organic matter. After reaching 500 °C, the temperature was further increased to 600 °C, where the samples were maintained for 6 h. This extended exposure to high temperatures was essential for the complete combustion of remaining materials, thereby providing a precise measurement of ash content. Upon completion of the 6 h duration at 600 °C, the temperature was gradually reduced to 100 °C over a programmed period of 4 h. The whole temperature program is presented in Figure 1.

The gradual cooling allowed the samples to stabilize and preserve the structural integrity of the ash residues. Once cooled in the desiccator to room temperature, the samples were weighted. The final ash content was calculated based on the residual ash and compared to the initial dry weight of the samples.

#### 2.4.2. Glucose and Xylose Content

The glucose and xylose content in the miscanthus samples was determined using an acid hydrolysis method involving 72% sulfuric acid (H_2_SO_4_), following the protocols established by Christakopoulos et al. (1993) [26], and Solár et al. (2011) [27]. The procedure began by preparing an extractive-free material to eliminate non-structural components, such as waxes, resins, and other soluble extractives, which could interfere with the carbohydrate analysis. The material was subjected to ethanol extraction for 10 h in a Soxhlet apparatus. The extractive-free material was treated with 72% H_2_SO_4_ under controlled conditions. The sulfuric acid treatment was conducted at 30 °C for 1 h. It was designed to break down the complex lignocellulosic matrix, disrupting the tightly bound cellulose and hemicellulose structures and making them more accessible for further hydrolysis. The initial treatment was followed by diluting sulfuric acid with water to a concentration of 3%. The dilution step prevented excessive degradation of the sugars into by-products, such as furfural and hydroxymethylfurfural (HMF). After dilution, the mixture was heated to its boiling point and maintained at that temperature for 4 h. The liquid phase, containing the hydrolyzed sugars, was carefully separated from the solid residues after this time. This liquid phase was used to determine the concentrations of carbohydrates-glucose and xylose. By following this method, the structural carbohydrate content of miscanthus could be accurately quantified, providing insight into the understanding of the carbohydrate composition of the biomass after HWE treatment.

The samples were neutralized to a pH of about five before HPLC analysis. The samples were centrifugated for 10 min at 12,000 rpm. Following centrifugation, each sample was filtered through a nylon syringe filter with a 0.2 μm porosity to remove any remaining particulates. Analysis of glucose and xylose in the hydrolysate was performed using a Shimadzu liquid chromatograph consisting of a CBM-20A control module, RID-10A refractometer detector, CTO-20A oven, DGU-20A degasser, and LC-20AD pump. Chromatograms were prepared using LC Solution v.1.21 SP1 software. For acid-hydrolyzed samples, the conditions were as follows:

- eluent: redistilled water;
- column: Rezex™ RHM-Monosaccharide H+ Phenomenex;
- oven temperature: 80 °C;
- flow rate: 0.6 mL·min^−1^;
- injection volume: 20 µL.

The calibration solutions for glycose and xylose were diluted using the following concentrations: 4.16, 2.08, 1.39, 1.04, 0.46, and 0.38 mg·cm^−3^. Equations of calculated calibration curves for xylose and glucose were as follows:

- y = 3.184^−10^ × x − 3.270^−5^; *R*^2^ = 0.9985 (xylose);
- y = 7.249^−10^ × x − 4.553^−4^; *R*^2^ = 0.9964 (glucose).

#### 2.4.3. Lignin Content

Lignin content was analyzed in native miscanthus and after the first, second, and third hot water extraction (HWE) cycles to investigate how repeated extractions impact lignin composition. The lignin was divided into two main fractions: acid-insoluble lignin (AIL) and acid-soluble lignin (ASL). The AIL was determined by subjecting the material to acid hydrolysis, after which the solid residue, representing the undissolved lignin, was collected and weighed. This fraction indicates the portion of lignin that remains intact and resistant to hydrolysis. The ASL was measured by analyzing the liquid obtained after acid hydrolysis, where the dissolved lignin fraction was quantified using UV-VIS spectrophotometry at a wavelength of 240 nm. This soluble lignin fraction represents lignin that breaks down and dissolves during the acid treatment, giving insights into the degradability of lignin during the HWE process. NREL/TP-510-42618 conducted the ASL and AIL measurements according to NREL’s standardized protocols. The method involves multiple steps, including the preparation of the miscanthus samples, the application of acid hydrolysis, and the subsequent analysis of both solid residues and the liquid.

### 2.5. Statistical Properties

The statistical analysis used an Analysis of Variance (ANOVA) to assess the significance of differences observed in the data. ANOVA is a powerful statistical tool that allows researchers to determine if there are any statistically significant differences between the means of studied groups. Following the ANOVA, a post hoc Duncan’s test was employed. Duncan’s test aimed to identify which specific groups were statistically distinct from one another. This allowed for a more nuanced understanding of the results beyond knowing the differences. In the context of this study, Duncan’s test is a statistical method used to compare multiple groups after an ANOVA. The reduction helps reduce the chance of incorrectly concluding differences between groups when there are no differences. By applying Duncan’s test, the analysis can pinpoint the groups that differed significantly, providing valuable insights into the relationships between the variables under investigation.

## 3. Results

### 3.1. Physical Material Characteristic

**Figure 2:**
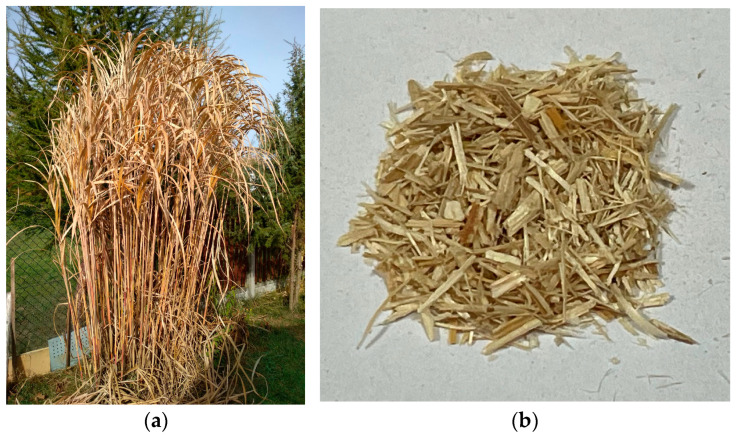
The *Miscanthus Gigantus*; (**a**) stalks, (**b**) fracture structure.

An evaluation of moisture content and granulometric composition of prepared plant samples (*Miscanthus Gigantus*) was performed in laboratory tests. Reference [28] indicates that the above parameters directly affect material compaction, which needs to be optimized under controlled laboratory conditions. Grochowicz and his team [29] analyzed the compaction process of lupin straw, where the material’s moisture content is 13–14%. Grass compaction at similar moisture contents from 12 to 15% was also studied by another team of researchers [30], determining the density of pellets made from shredded wheat straw, barley straw, maize hulls, and switch millet. When log bran was compacted, an increase in moisture content decreased the quality of the resulting pellets [31]. The study found that the moisture content in this range positively influenced the compaction process of all materials tested. Based on the results, the best value was achieved by compacting shredded maize husks with a moisture content of 12%, which resulted in a density of 1136 kg∙m^−3^. Considering the above literature data, it is believed that the most suitable value of material moisture content for the compacting process should range from 10% to 15%. The fracture structure of *Miscanthus Gigantus* is presented in Figure 2.

The possibility of improving the quality and desirable properties of an agglomerate by adjusting the physical characteristics of the raw material is a crucial aspect in the context of biofuel and bioproduct production. Compaction processes consume considerable energy, which may impact the feasibility of products from biomass, such as pellets from *Miscanthus giganteus*. The compacting process may increase density and facilitate biomass transportation, but it can negatively affect the lignocellulosic structure, for example, in subsequent cellulose extraction processes [6,32]. Due to high temperature and pressure, cellulose is degraded during compacting, making extraction less efficient [33]. Optimizing the compacting parameters, however, can lead to benefits such as biomass pretreatment, facilitating subsequent stages in the cellulose production process [34]. The relationship between compacting and cellulose production efficiency still needs to be better understood to develop sustainable strategies for using biomass such as *Miscanthus giganteus* to generate biofuels and other bioproducts. [35].

### 3.2. Moisture and Sludge Mass in the HWE Process

The *Miscanthus Gigantus* samples were analyzed in their raw form after being treated with hot water extraction (HWE). Analysis revealed that HWE treatment significantly affected miscanthus sample moisture content. Raw and untreated material contained a shallow moisture content, averaging around 0.5%, typical of dried biomass feedstocks. As a result of the HWE treatment, the moisture content of the biomass significantly increased, indicating that the therapy penetrated the biomass structure and allowed water to be absorbed. As moisture content increased with each HWE cycle, the moisture level rose. The biomass was initially saturated based on the moisture content in the first HWE cycle. In the second cycle of HWE treatment, the moisture content increased, indicating water uptake continued. The third HWE cycle yielded the highest moisture content, suggesting a near-saturation point or potential structural changes in the biomass. The substantial increase in moisture content following HWE treatment illustrates the capacity of biomass to absorb water, particularly once multiple extraction cycles have been performed. Elevated moisture levels may affect subsequent processing steps, such as palletization and cellulose extraction, as they can affect energy efficiency and effectiveness. As a result, the observed moisture increase post-HWE treatment highlights a crucial factor to consider when developing biofuels and bioproducts using this technique—the moisture of raw material and after the HWE process are presented in Table 1.

According to the table, hydrothermal treatment (HWE) significantly increases the ability of lignocellulosic materials to absorb water, increasing moisture content. A study on oat grain showed that HWE modified its internal structure, increasing its ability to bind water [36]. Similar effects were observed with triticale, where an increase in grain moisture caused a decrease in specific, bulk, and shaken density [37]. After HWE, despite increasing moisture content to 22.105% by weight, the density of the compacted terms remained unchanged. The additional moisture may have acted as a plasticizer, facilitating particle deformation during compaction and compensating for the potential reduction in density due to the higher moisture content. The same effect was observed in the study of oat grain, where hydrothermal treatment increased the grain’s capacity to absorb water [36]. Based on the literature, HWE can increase the moisture content of lignocellulosic materials and affect the density after compaction by changing their physical properties.

**Figure 3:**
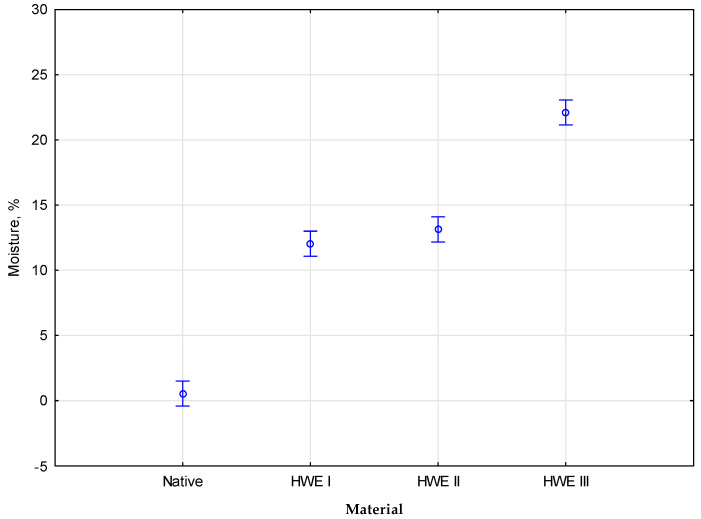
The coloration between moisture and modification type.

The *Miscanthus Gigantus* samples were used for compaction analysis in their raw form after being treated with hot water extraction (HWE). Compared with other raw materials, the moisture content was shallow, at an average level of 0.5%. The low moisture level can significantly affect the compaction process as well as the properties of the resulting pellets. In the HWE treatment, miscanthus was exposed to high-pressure and high-temperature water for three consecutive cycles to modify the materials’ properties, potentially affecting their compaction capabilities. Based on the differences between raw material and the cycles of HWE I, HWE II, and HWE III, the cumulative effects of the treatment on material characteristics and subsequent compaction could be investigated. The final compaction study examined the impact of these HWE-induced changes on granulation and pellet quality. The study focused on pellet density to determine their energy content and suitability for use as biofuels. The data collected for the study were analyzed using statistics, allowing the types of modifications of HWE to be identified. According to the statistical analysis, the measured parameters differed significantly. As a result of the empirical statistic *F*(3, 8) = 449.43, the results determine that the degree of significance is under 0.05. The coloration between moisture and modification type of material is presented in Figure 3.

Duncan’s test, which performed the additional post hoc analysis, showed that none of the parameters were homogeneous. According to the statistical analysis of the influence parameters, the degree of significance was significant. According to the alpha significance level, *p* was below the significance threshold of 0.05. The test indicates that all parameters belong to various homogenous groups. Statistically significant differences were found between the various modifications and moisture in HWE.

### 3.3. Material Compaction

Compaction was used to evaluate the mechanical and physical properties of pellets produced at 22 °C. The study systematically analyzed key parameters, such as pellet density, moisture content, and energy consumption, to determine which conditions enhance compaction efficiency. This research determined optimal conditions for minimizing energy consumption while improving the mechanical integrity of final products by understanding the relationship between moisture levels and density. Compaction process optimization and developing bio-based materials with tailored properties are important for meeting both sustainability goals and performance requirements. The process stops when compacting tension occurs at 6.5 MPa. Characteristics of the technical parameters and densities of compacted material are presented in Table 2.

There was a significant difference in density between cycles of HWE-treated miscanthus biomass after being compacted. A peak density of 388.7 kg∙m^−3^ was achieved following the first HWE cycle, but subsequent cycles recorded densities of 297.4 kg∙m^−3^ and 223.2 kg∙m^−3^, respectively, indicating gradually decreasing compaction efficiency. According to this trend, while the initial cycle optimizes particle packing, prolonged extraction may alter the material composition, reducing structural compactness. The hydrothermal treatment of lignocellulosic materials increases their compaction efficiency and increases their density. In the HWE process, hemicelluloses and cellulose are partially decomposed, which makes the material more plastic and facilitates compaction. The HWE process also removes some mineral components, which reduces the structure’s rigidity and improves the particles’ packing during compaction. Thus, materials treated with HWE have a higher density after compaction than materials not treated with HWE. The energy density of biomass after hydrothermal carbonization increased after hydrothermal treatment [38].

**Figure 4:**
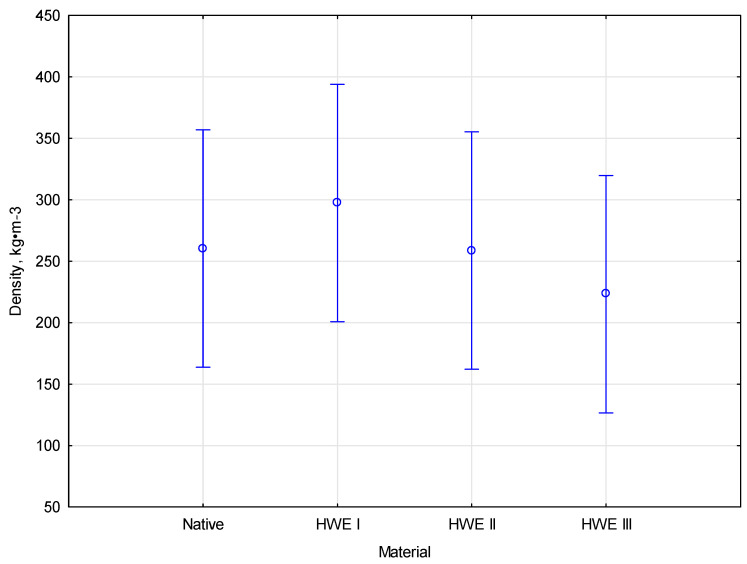
The coloration between density and modification type.

Compaction is more efficient when particles are packed better using the HWE process, possibly due to modifications in the material’s composition or structure. In contrast, raw materials without pre-treatment exhibited the lowest densities, falling below 278.5 kg∙m^−3^. The importance of pre-treatment or modification steps in optimizing the compaction process and achieving desired material properties can be seen in this observation. In order to maximize the final product’s density and associated characteristics for specific applications, compaction process parameters must be controlled with care and understood. The study data were statistically analyzed in order to identify the types of modifications of HWE in camper to deniability. The statistical analysis showed no significant differences between the measured parameters. The empirical statistic *F*(3, 8) = 0.523 determined that the *p*-value equals 0.679. The coloration between density and modification type of material is presented in Figure 4.

The analysis of Duncan’s test revealed homogeneity between all parameters, indicating no statistically significant differences between them. There is no significance in the statistical analysis, which confirms this conclusion. The alpha significance level of 0.05 further confirms the finding that all parameters belong to homogenous groups. Based on this result, any observed differences between the parameters are likely the result of random chance. Due to their statistical equivalence, these parameters can be treated as a single parameter.

### 3.4. Compaction Energy Consumption

#### 3.4.1. Compaction Process

**Figure 5:**
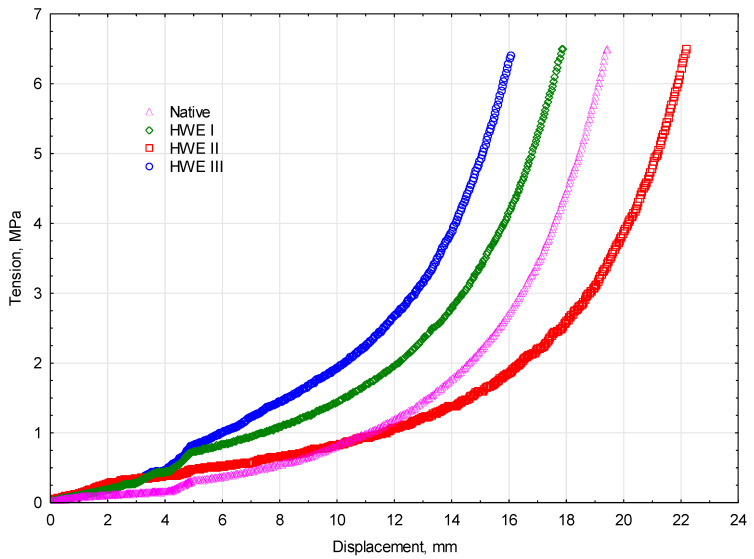
The characteristics of the *Miscanthus Gigantus* compaction process.

Energy consumption during the entire process of compacting is a function of the work performed during that process. The study found that the particle size of the material affects the amount of energy consumed during a compressive process in a chamber with a diameter of 30 mm. Using a steel cylindrical sleeve encased in a head, the prototype attachment compacted the material. Compaction occurs in several phases, each of which determines the overall energy consumption, underscoring the need to optimize individual steps. The smaller the particles, the greater the amount of energy it takes to compress them. Using force, the material was pressed down on the prototype device, removing the air within the sleeve. In order to optimize the compaction process for energy efficiency, this relationship needs to be understood. The compaction graph is analyzed to determine how much energy is used in the process. The graph provides valuable information about the relationship between process parameters, such as compaction force or duration, and energy consumption. Optimization can be made to increase the energy efficiency of the process. The characteristics of the compaction process of miscanthus for raw material and different types of modification treatments are presented in Figure 5.

The results of the experiments indicate that compacting material with diverse HWE treatments requires less energy than compacting raw material. Previous studies have explored the influence of process parameters on compaction energy consumption. The regression model developed by Hu et al. [39] predicts energy consumption from rice straw compaction experiments. Skonecki and Laskowski (2012) [40] state that reducing moisture content and chamber diameter decreases energy consumption during wheat straw compaction. According to their study, reducing moisture content from 18% to 10% or decreasing chamber diameter from 18 mm to 12 mm significantly reduced total energy consumption. In the case of a chamber diameter of 18 mm and a moisture content of 10%, the energy consumption was 85.7 J, while in the case of a chamber diameter of 12 mm and a moisture content of 18%, it was 33.8 J. Based on Skonecki’s [40] study of miscanthus sugarcane meal and Pennsylvania sida, the observed trend of reduced energy consumption with reduced moisture content and chamber size can be explained.

#### 3.4.2. Compaction Energy Expenditure

The studies results provided valuable insight into the material behavior of *Miscanthus Gigantus* under stress. Based on the calculation of the energy values [41] for lignocellulosic raw materials, it was possible to characterize the effect of inorganic compounds and material properties during the process. The characterization provides some insight into the optimal properties of the raw material during compaction. Trigonometric functions were fitted to individual thickening runs to measure the work performed during the thickening process. It was calculated using least squares to estimate the trend line’s range, and the coefficient of determination, *R*^2^, confirmed that each time the fit exceeded 0.99, confirming the high quality of the fit.

In most cases, a third-degree polynomial was fitted to determine pressure change. Through this action, the assumption was realized that the quality of the fit would be maintained. The obtained polynomial was integrated based on the data to determine the energy value of the process. The integral equation, the coefficients, the density value, and instructions for evaluating the coefficients for specific parameters are presented in Table 3.

The total work in the compaction process was calculated using shredded miscanthus material. The total work performed during the compaction process, based on displacement, ranges from 2.800 × 10^−5^ J to 4.958 × 10^−5^ J. There are differences in energy requirements based on the HWE modification, which indicates that the number of cycles influences the energy requirements. According to the analysis, a third-degree polynomial was the best choice for describing compaction forces. The coefficient of determination *R*^2^ for the theoretical model and the experimental data were very close, indicating a very high level of accuracy. It confirms the validity of the fit and the stability of the compaction process when the coefficient of determination *R*^2^, which measures the fit of the theoretical model to the experimental data, oscillates between 0.991 and 0.995. Estimating the mechanical and operational properties of the compaction process was essential for assessing its efficiency. Based on these results, fine-tuning the HWE method’s production parameters against cycles is possible.

**Figure 6:**
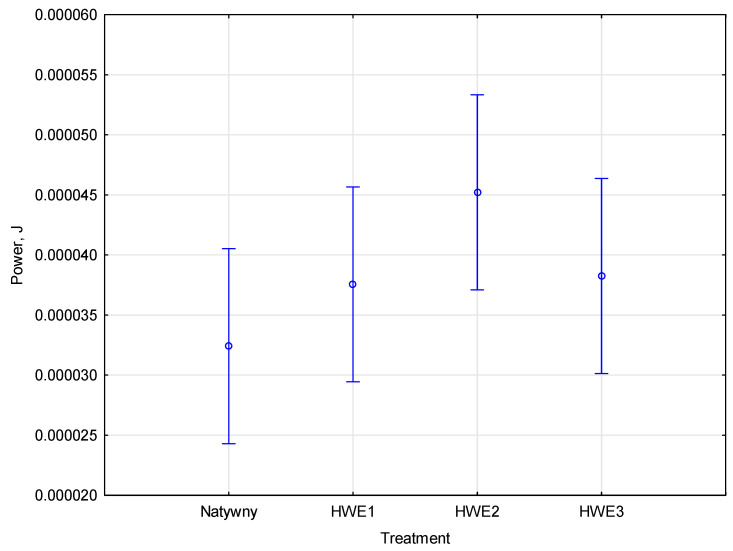
The total compaction work compared and modification type.

The analytical study examined the effect of HWE modification on the compaction process and energy intensity of the prepared material. Based on the study’s results, it was possible to identify which HWE modification cycle affects the product manufacturing process and the energy spent during raw material compaction. To assess differences between factors and their strength of influence, a one-way analysis of variance (ANOVA) was used. Based on ANOVA and Duncan’s test statistics, it was found that the two groups spent different amounts of energy during compaction. The results determine a degree of significance on the level *p* = 0.16139, which is higher than the value of 0.05. Duncan’s test allowed the identification of homogeneous groups and detailed comparisons of average energy expenditure values. According to the empirical statistic, the current effect equals *F*(3, 8) = 2.2362. Optimizing mixture compositions can reduce energy consumption when compacting. Identifying specific parameters that affect the energy efficiency of the process can be the focus of further research. The total compaction work compared to the number of HWE cycles is presented in Figure 6.

The prepared sample was divided based on the number of native and HWE cycles. Based on how many HWE cycles are performed, Miskantus material compaction partially affects energy expenditure. The total compaction work for a mixture of native material that was not modified was 2.800 × 10^−5^ J. Native material and material modified by cycles one and two of HWE were in the same group. The final material sample was subjected to three HWE cycles and produced a total work value of 3.470 × 10^−5^ J. Duncan’s test detailed the differences between groups to optimize the manufacturing process of compacting the subjected material. The effect of miscanthus modification state on energy expenditure enables adjustment of these parameters. Duncan’s test can be beneficial for identifying practical differences between groups. The results are helpful in refining process parameters and modifying miscanthus for specific applications.

### 3.5. Sludge After Hot Water Extraction (HWE)

An extraction process with hot water was conducted on the prepared test material. The HWE process involved interfering with a liquid medium into the structure of the test material to extract compounds. Compounds that are solvent-soluble are more readily extracted at an elevated temperature. According to the process, partial degradation of hemicellulose leads to precipitates forming. Suppose the resulting precipitate contained hemicellulose and lignin with low molecular weights after degradation. Approximately 10 g of raw material was treated with the HWE process. Different HWE steps may have different effects on the material and its mass. The mass of the residue containing 400 mL distilled water evaporation in a particular reactor was obtained from HWE. The sludge mass-produced by the HWE process after distilled water in the first cycle was equal to 0.108 g, with a standard deviation of 0.043 g. In cycle HWE II, the value was 0.032 g with a standard deviation of 0.030 g, and the last HWE III was equal to 0.043 g, where the standard deviation was 0.031 g.

To estimate the mass of sludge remaining after the process in the reactor, a hot water extraction was carried out, and a three-cycle HWE process was repeated for each HWE I, HWE II, and HWE III cycle. The sludge mass was measured three times per cycle. Based on the results, the first HWE cycle produced the highest sludge mass, and the second cycle produced the lowest. The first extraction cycle had the most excellent effectiveness in removing substances from the material, while the subsequent cycles were characterized by decreased efficiency in removing additional components. The process may have removed the most readily available compounds in the first phase, leaving the harder-to-extract substances behind in the second phase. The results may help optimize extraction processes, adjusting parameters or cycle numbers to achieve the desired degree of component removal and maintain cost and time efficiency.

### 3.6. Chemical Composition

#### 3.6.1. Ash Content

The ash content of a material refers to the amount of inorganic residue that remains after combustion. The reduction in ash content is essential in some industries (such as biomass processing and biofuels) to improve the material’s quality, fuel efficiency, or processing characteristics. For example, controlling ash content is crucial for optimizing combustion processes and enhancing the overall sustainability of energy production. Lower ash content can reduce equipment wear and maintenance costs and lower emissions of harmful pollutants. The ash content of miscanthus before and after multiple HWE cycles is presented in Table 4.

**Figure 7:**
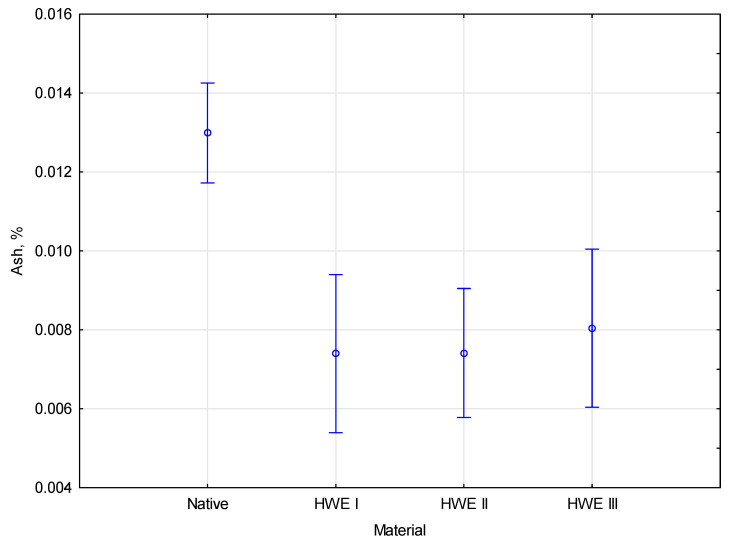
The coloration between ash content and modification type.

After the first HWE cycle, the ash content was reduced from 1.3% to 0.7%, lowering the ash content by almost 50%. The ash content remained similar after the second and third HWE cycles but was still lower than the native value. This suggests that part of the ash is removed in the first cycle and that subsequent cycles have a minimal effect on further decreasing the ash content. In addition, due to the weight loss of the material after all HWE cycles, the ash content relative to the original weight of the material would be even lower. This reduction in relative ash content further enhances the material’s suitability for applications where low inorganic residue content is critical, such as biofuel production. As the material becomes more refined in the HWE process, its performance characteristics, such as combustion efficiency and overall quality, will likely improve. A statistical analysis of the data were conducted, and the results showed that the *F*(3, 8) value was 18.920, which determined a *p*-value of 0.00054. Based on the statistical analysis, only one parameter showed a significant difference in competition. The coloration between ash content and the modification type of material is presented in Figure 7.

According to post hoc analysis, one of the parameters was homogeneous. The test indicates that all parameters belong to different homogeneous groups. Only one statistically significant difference existed between the modifications and ash after HWE. In light of these findings, future research should examine the factors contributing to the observed statistically significant difference.

#### 3.6.2. Lignin Content

Lignin content was evaluated for native miscanthus following the first, second, and third hot water extraction (HWE) cycles to examine the effects of repeated extractions on lignin composition. The lignin was separated into two primary fractions: acid-insoluble lignin (AIL) and acid-soluble lignin (ASL). Analyzing lignin content during HWE cycles revealed nuanced changes. It was observed that the acid-insoluble lignin fraction (AIL) showed a slight decrease after the first cycling, stabilizing in subsequent cycles. In contrast, the acid-soluble lignin fraction (ASL) increased after the first treatment, suggesting partial lignin breakdown. Based on these changes, HWE modifies the chemical matrix of biomass, which may reduce enzymatic accessibility downstream. This distinction allows a more detailed understanding of how lignin behaves during extraction—the lignin content in native and after HWE treatment miscanthus is presented in Table 5.

**Figure 8:**
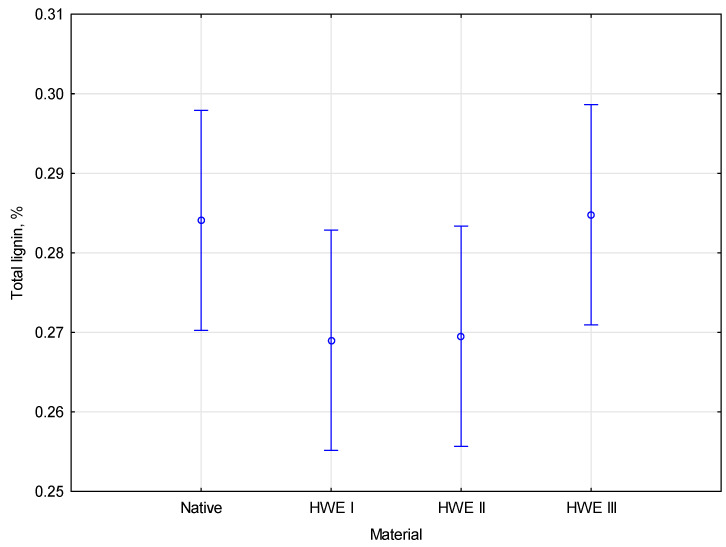
The coloration between lignin content and modification type.

In native material, the total lignin content is 28.4%, with 3.2% coming from ASL and 25.2% from AIL. The total lignin content decreases slightly after the first two cycles (about 5%) compared to the native material. After the first HWE cycle, the total lignin content slightly decreases to 26.9%, with a reduction in both ASL and AIL. After the second HWE cycle, the total lignin content remains relatively stable at 27.0%, with ASL at 3.0% and AIL at 23.9% compared to the miscanthus after the first HWE cycle. The total lignin content returns to its original value (28.5) after the third HWE cycle. The data suggests that the ASL to AIL ratio remains constant across all HWE cycles, indicating that the relative distribution of soluble and insoluble lignin fractions does not change significantly. Most of the reductions in lignin content occur during the first HWE cycle, with limited changes in subsequent cycles. The HWE process appears effective at removing ash from the material while having a minimal impact on the lignin content. Statistical analysis of the lignin content allowed the identification of the types of modifications to HWE. Statistical analysis revealed no significant differences between the measured parameters. Using the empirical statistic *F*(3, 8), *p* = 0.17457, we determine that the degree of significance is below 0.05. The coloration between lignin content and the modification type of material is presented in Figure 8.

According to Duncan’s test, the parameters were homogeneous after the additional post hoc analysis was performed. The degree of significance was based on the statistical analysis of the influence parameters. Using the alpha significance level, *p* was over the significance threshold 0.05. This indicates that all parameters could be classified as one homogenous group.

#### 3.6.3. Glucose and Xylose Content

Miscanthus is a lignocellulosic biomass that contains significant amounts of fermentable sugars, mainly glucose and xylose, making it a valuable feedstock for biofuel production. The composition of the sugars can be affected by various processing methods, such as hot water extraction (HWE), which can selectively remove hemicellulose and affect the availability of these sugars. Optimizing the extraction process is essential to maximize sugar yields and increase the efficiency of converting miscanthus into renewable energy sources. Measuring glucose and xylose can provide valuable information on the material’s composition, properties, and applicability. Precise analytical methods, such as chromatography, allow the quantitative determination of glucose and xylose, which provides for quality control in the context of furthering knowledge of the materials under study—the glucose and xylose content in native and after HWE treatment miscanthus is presented in Table 6.

For the native material, the glucose content is 66.8%, and the xylose content is 28.3%. After the first HWE cycle, the glucose content in miscanthus decreases by about 5% to 63.7%, while the xylose content stays at the same level (28.6%). After the second HWE cycle, the glucose content in miscanthus rebounds to 65.8%, while xylose slightly decreases to 27.5%. After the third HWE cycle, the glucose and xylose content remains stable compared to the miscanthus after the second HWE cycle. Variations in glucose and xylose after successive cycles of HWE are relatively small, suggesting that HWE has a limited effect on extracting these sugars from the material.

Variations in glucose and xylose after successive cycles of HWE are relatively small, suggesting that HWE has a limited effect on extracting these sugars from the material. The HWE process was carried out at 100 °C. The structural components of the wood are resistant to such temperatures. The least thermally resistant are hemicelluloses, which, among other things, have a lower average degree of polymerization than cellulose. Their thermal degradation begins at temperatures higher than 100 °C. Hot water can cause the leaching of only monosaccharides and selected oligomers from biomass.

**Figure 9:**
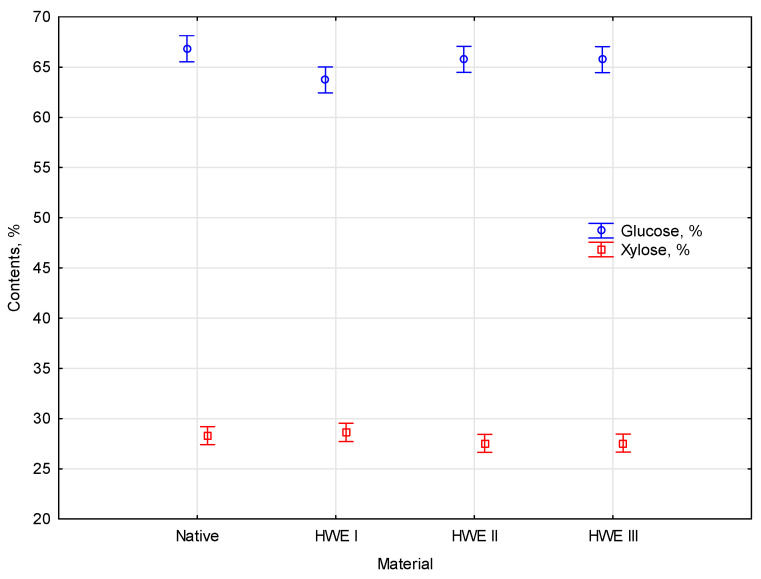
The coloration between glucose and xylose contents contrasts modification type.

There were no significant changes in lignin (ASL and AIL), or in the glucose and xylose levels, in the studied chemical composition of miscanthus. This shows that exposure to high temperature and elevated pressure in an aqueous environment does not affect the content of structural components but mineral substances. This is a desirable effect, as it does not cause the loss of valuable components of lignocellulosic biomass. Other changes caused by the HWE process can also affect the structure of the material under study, e.g., causing an increase in porosity or loosening the bonding of the components of the lignocellulosic complex. Statistics were used to analyze the collected data, allowing the types of modifications to be identified. The measured parameters differed significantly according to the statistical analysis. An empirical statistic of *F*(6, 14) = 2.8534 with a *p*-value of 0.04969 was obtained. The coloration between glucose and xylose contents, in contrast to modification type of material, is presented in Figure 9.

Duncan’s test performed as a post hoc analysis showed no homogeneous parameters. Based on statistical analysis, the influence parameters were significant. Based on the test results, all parameters appear to fall into the same homogeneous group beside one. Different modifications and moisture in HWE showed the same statistically significant differences. The modification changed the value of glucose content in *Miscanthus giganteus*.

## 4. Discussion

The study demonstrated that HWE significantly affects the compaction efficiency and material properties of *Miscanthus giganteus*. For example, after a single HWE cycle, the density of compacted material increased to 388.7 kg·m^−3^, compared to 260.3 kg·m^−3^. Ash content decreased by 50% after the first cycle, demonstrating a clear improvement in material quality for biofuel applications. Energy consumption during compaction also declined significantly, suggesting improved efficiency. In comparison, studies by Grochowicz et al. (2014) [29] and Skonecki and Laskowski (2012) [40] have shown that similar pretreatments can enhance compaction efficiency in other lignocellulosic materials, such as wheat and barley straw. However, these studies reported slightly lower density gains than the current miscanthus results. The higher performance observed here may be attributed to miscanthus’s specific chemical and physical properties, including its lignin and hemicellulose content, which are particularly well-suited for HWE-based treatments. These findings support the hypothesis that HWE pretreatment offers a scalable method to enhance biomass quality and efficiency in pellet production, aligning with previous research while demonstrating superior results for *Miscanthus giganteus*.

Following the first HWE cycle, the ash concentration in the experiments decreased by nearly 50%, from 1.3% to 0.7%. Even after the second and third HWE cycles, the ash content was still below the original value. Depending on the level of extraction, the ash reduction for switchgrass varies from 20.7% to 69.6%, and for loblolly pine bark, it varies from 57.0% to 73.3%. Consequently, HWE considerably lowers the amount of ash [42]. After HWE, the ash percentage of willow biomass varied between 0.6% and 2.1% in winter storage heaps and between 2.1% and 3.4% in summer storage piles [43]. This suggests that HWE can lower ash content; however, storage circumstances will determine how much of an impact this has. HWE lowered Willow pellets’ ash level to less than 1%, equivalent to untreated maple biomass [44]. This implies that HWE effectively reduces the ash in biomass meant for pellet manufacture.

The overall lignin concentration in the native material is 28.4%, with ASL accounting for 3.2% and AIL for 25.2%. After the first two cycles, the total lignin concentration drops by around 5% compared to the original material. Both ASL and AIL drop after the first HWE cycle, resulting in a modest decrease in the overall lignin concentration to 26.9%. Compared to miscanthus after the first HWE cycle, the total lignin content stays comparatively constant at 27.0% after the second HWE cycle, with ASL at 3.0% and AIL at 23.9%. Following the third HWE cycle, the total lignin content reaches its initial value of 28.5%. HWE performed on softwood (yellow pine) at temperatures ranging from 140 to 320 °C revealed that lignin in the solid fraction gradually decreased as the temperature rose. At 260 °C, the most lignin-rich material (~4.2%) was produced [45]. Lignin content often decreases more at higher temperatures and longer times [45,46].

One technique for processing biomass is hot water extraction (HWE), frequently used to recover essential components like hemicelluloses, which contain xylose and glucose. The parent material in our investigation contained 28.3% xylose and 66.8% glucose. The xylose concentration of miscanthus stayed at 28.6% after the first HWE cycle. However, the glucose content dropped by almost 5% to 63.7%. Following the second HWE cycle, miscanthus’s xylose concentration marginally declined to 27.5%, but its glucose level rose to 65.8%. In contrast to miscanthus after the second HWE cycle, the amount of glucose and xylose was constant during the third HWE cycle. Although the process conditions differed, which could have contributed to this, a higher drop in xylose content was also seen. Additionally, there was a decrease in the amount of ash and slight variations in the amount of lignin [47]. HWE at 180 °C and 13 bar produced a glucose content in the hydrolysate of 6.70 g/L in the Saccharina japonica research [48]. When HWE was applied to maize stover for 210 min at 160 °C, 70.2% of the total xylan was dissolved, mainly producing xylooligosaccharides [49]. Longer extraction durations and higher temperatures often result in higher sugar yields, but they can also cause sugar to degrade into byproducts such as furfural [48,50]. Based on the experimental results, HWE treatment significantly affects miscanthus biomass density and lignin composition. The first HWE cycle offers the best balance between structural and chemical optimization for practical implementation. The aim of further studies could be to refine multi-cycle treatments to optimize both compaction efficiency and material modification.

## 5. Conclusions

The hot water extraction process (HWE) significantly impacted the characteristics of miscanthus biomass, providing a foundation for optimizing biofuel production. After three treatment cycles, the moisture content of the biomass increased dramatically from 0.5% to 22% due to the HWE process, demonstrating the material’s remarkable capacity to absorb water under high pressure and temperature. In addition, during the initial HWE cycle, the material’s density rose by around 40%, peaking at 388.7 kg/m^3^. This implies improved biomass particle packing following treatment. As a result of the partial breakdown of hemicellulose caused by the HWE process, precipitates were also produced. During the initial treatment cycle, the mass of these precipitates peaked at an average of 0.108 g ± 0.043 g and subsequently gradually declined. As an additional benefit of the HWE process, the ash content of the biomass was reduced by 50% during the initial cycle, making it suitable for biofuel production applications that require low mineral content.

After the first HWE cycle, lignin levels somewhat decreased, according to lignin content analysis; however, following cycles, the value recovered to baseline. In contrast, the xylose content was nearly constant following the first cycle, whereas the glucose amount was significantly reduced. In later cycles, there were slight variations in the amounts of both sugars. In general, the characteristics of *Miscanthus giganteus* biomass are affected in several ways by HWE treatment. On the one hand, it causes the material’s density and moisture content to rise while its ash content decreases; on the other hand, it alters the chemical makeup of the biomass, leading to partial hemicellulose breakdown, among other things. This study demonstrates that HWE can be used to improve the quality of miscanthus biomass for use in the production of biofuels and other industrial processes. HWE may be able to optimize pelletization, cellulose extraction, and biofuel synthesis in the future as a result of the findings from this study. In future studies, these methods could be scaled up for industrial applications and integrated into biofuel production chains to assess economic feasibility.

## Figures and Tables

**Table 1:** The moisture of raw material and after the HWE process.

Material	Mass Before Drying (SD), g	Mass After Drying (SD), g	Moisture (SD), %
Native	28.115 (4.732)	27.964 (4.725)	0.547 (0.067) ^a^
HWE I	186.725 (0.902)	166.652 (1.005)	12.047 (0.777) ^b^
HWE II	195.275 (1.232)	172.612 (0.459)	13.131 (0.979) ^b^
HWE III	173.376 (1.987)	141.986 (0.818)	22.105 (0.721) ^c^

SD—standard deviation; ^a, b, c^—homogeneous groups.

**Table 2:** Characteristics of the technical parameters and densities of compacted material.

Material	Mass, g	Height, mm	Volume, mm^3^	Density, kg∙m^−3^
Native	0.533 (0.048)	13.333 (1.247)	2051.467 (191.897)	260.330 (12.904) ^a^
HWE I	0.587 (0.124)	13.333 (2.357)	2051.467 (362.651)	297.384 (83.785) ^a^
HWE II	0.596 (0.238)	15.000 (4.082)	2307.900 (628.131)	258.677 (64.376) ^a^
HWE III	0.573 (0.242)	7.000 (2.944)	2461.760 (452.952)	223.232 (51.987) ^a^

^a^—homogeneous group.

**Table 3:** The calculations of total compaction work using the integral equation.

Total Work Carried out Under Specified Conditions *W_(τ, φ)_*	Determination Coefficient *R*^2^ (SD)	Displacement l (SD), mm	Total Compaction Work, J (SD)
$W_{\left(Native\right)}=\int_{0}^{0.001\cdot l}0.0013x^{3}-0.0212x^{2}+0.12x$	0.991 (0.003)	21.628 (1.840)	2.800 × 10^−5^ (3.206 × 10^−6^) ^a^
$W_{\left(HWE\;I\right)}=\int_{0}^{0.001\cdot l}0.0014x^{3}-0.0234x^{2}+0.1803x$	0.994 (0.006)	20.784 (2.100)	3.887 × 10^−5^ (6.986 × 10^−6^) ^a,b^
$W_{\left(HWE\;II\right)}=\int_{0}^{0.001\cdot l}0.0011x^{3}-0.0235x^{2}+0.185x$	0.993 (0.008)	23.173 (2.488)	4.958 × 10^−5^ (5.760 × 10^−6^) ^a,b^
$W_{\left(HWE\;III\right)}=\int_{0}^{0.001\cdot l}0.0014x^{3}-0.0223x^{2}+0.1757x$	0.995 (0.006)	19.892 (1.349)	3.470 × 10^−5^ (1.054 × 10^−5^) ^b^

SD—standard deviation; ^a, b^—homogeneous groups.

**Table 4:** Ash content in native miscanthus and after the HWE process.

Material	Ash (SD), %
native	1.3 (0.2) ^a^
after Ist HWE cycle	0.7 (0.1) ^b^
after IInd HWE cycle	0.7 (0.1) ^b^
after IIIrd HWE cycle	0.8 (0.1) ^b^

SD—standard deviation; ^a,b^—homogeneous group.

**Table 5:** Lignin content in native and after HWE treatment miscanthus.

Material	ASL (SD), %	AIL (SD), %	ASL/AIL	Total Lignin (SD), %
native	3.2 (0.2)	25.2 (1.2)	0.13	28.4 (1.4) ^a^
after Ist HWE cycle	3.0 (0.2)	23.9 (1.0)	0.13	26.9 (0.8) ^a^
after IInd HWE cycle	3.0 (0.4)	23.9 (1.0)	0.13	27.0 (0.7) ^a^
after IIIrd HWE cycle	3.3 (0.4)	25.2 (0.7)	0.13	28.5 (1.0) ^a^

SD—standard deviation; ^a^—homogeneous group.

**Table 6:** Glucose and xylose content in native and after HWE treatment miscanthus.

Material	Glucose (SD), %	Xylose (SD), %
native	66.8 (1.1) ^a^	28.3 (0.6) ^c^
after Ist HWE cycle	63.7 (0.6) ^b^	28.6 (0.4) ^c^
after IInd HWE cycle	65.8 (1.0) ^b^	27.5 (0.7) ^c^
after IIIrd HWE cycle	65.8 (1.0) ^b^	27.6 (0.9) ^c^

SD—standard deviation; ^a,b,c^—homogeneous groups.

## Data Availability

The original contributions presented in this study are included in the article. Further inquiries can be directed to the corresponding author.
